# Using synthetic biology to increase nitrogenase activity

**DOI:** 10.1186/s12934-016-0442-6

**Published:** 2016-02-20

**Authors:** Xin-Xin Li, Qi Liu, Xiao-Meng Liu, Hao-Wen Shi, San-Feng Chen

**Affiliations:** Key Laboratory for Agrobiotechnology and Key Laboratory of Soil Microbiology of Agriculture Ministry, China Agricultural University, Yuanmingyuan West Road No. 2, Haidian District, Beijing, 100193 People’s Republic of China

**Keywords:** Nitrogenase, *Paenibacillus*, Fe–S cluster assembly, Electron transporter

## Abstract

**Background:**

Nitrogen fixation has been established in protokaryotic model *Escherichia coli* by transferring a minimal *nif* gene cluster composed of 9 genes (*nifB*, *nifH*, *nifD*, *nifK*, *nifE*, *nifN*, *nifX*, *hesA* and *nifV*) from *Paenibacillus* sp. WLY78. However, the nitrogenase activity in the recombinant *E. coli* 78-7 is only 10 % of that observed in wild-type *Paenibacillus*. Thus, it is necessary to increase nitrogenase activity through synthetic biology.

**Results:**

In order to increase nitrogenase activity in heterologous host, a total of 28 selected genes from *Paenibacillus* sp. WLY78 and *Klebsiella oxytoca* were placed under the control of *Paenibacillus nif* promoter in two different vectors and then they are separately or combinationally transferred to the recombinant *E. coli* 78-7. Our results demonstrate that *Paenibacillus suf* operon (Fe–S cluster assembly) and the potential electron transport genes *pfoAB*, *fldA* and *fer* can increase nitrogenase activity. Also, *K. oxytocanifSU* (Fe–S cluster assembly) and *nifFJ* (electron transport specific for nitrogenase) can increase nitrogenase activity. Especially, the combined assembly of the potential *Paenibacillus* electron transporter genes (*pfoABfldA*) with *K. oxytocanifSU* recovers 50.1 % of wild-type (*Paenibacillus*) activity. However, *K. oxytocanifWZM* and *nifQ* can not increase activity.

**Conclusion:**

The combined assembly of the potential *Paenibacillus* electron transporter genes (*pfoABfldA*) with *K. oxytocanifSU* recovers 50.1 % of wild-type (*Paenibacillus*) activity in the recombinant *E. coli* 78-7. Our results will provide valuable insights for the enhancement of nitrogenase activity in heterogeneous host and will provide guidance for engineering cereal plants with minimal *nif* genes.

**Electronic supplementary material:**

The online version of this article (doi:10.1186/s12934-016-0442-6) contains supplementary material, which is available to authorized users.

## Background

Most biological nitrogen fixation is catalyzed by the molybdenum nitrogenase enzyme. The molybdenum nitrogenase is composed of two proteins, MoFe protein (NifDK) and Fe protein (NifH). The MoFe protein is an α_2_β_2_ heterotetramer that contains the iron–molybdenum cofactors (FeMo-co) and P clusters. The FeMo-co is a [Mo–7Fe–9S–C-homocitrate] cluster which serves as the active site of nitrogen binding and reduction. The P-cluster is a [8Fe–7S] cluster which shuttles electrons to the FeMo-co. The Fe protein is a γ_2_ homodimer bridged by an intersubunit [4Fe–4S] cluster that serves as the obligate electron donor to the MoFe protein [1–5].

**Fig. 1:**
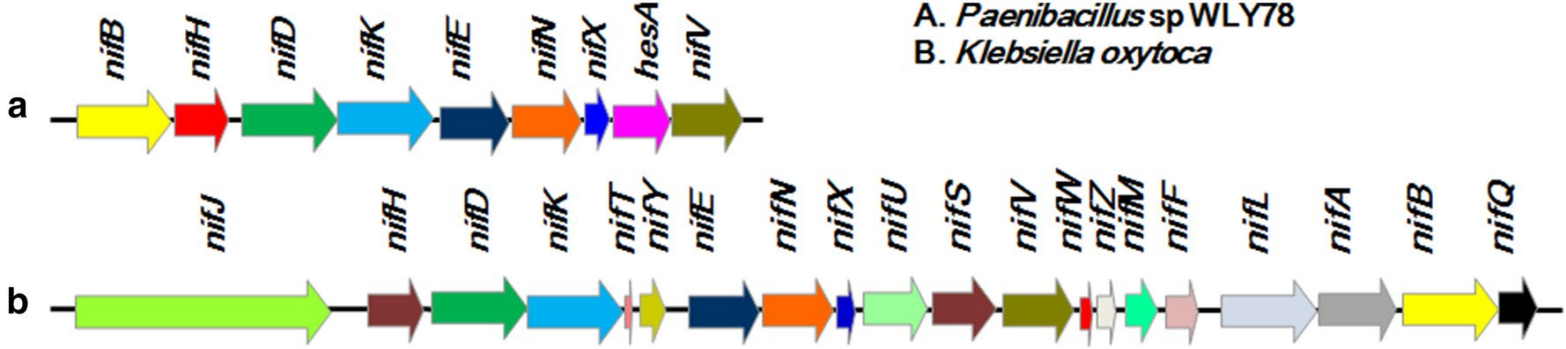
Schematic representation of the *Paenibacillus nif* gene cluster compared with syntenic *nif* clusters of *K. oxytoca* M5a1. **a** *Paenibacillus* sp. WLY78. **b** *K. oxytoca* M5a1

Although the biochemical properties and structure of molybdenum nitrogenases are remarkably similar when purified from diverse bacteria and archaea, the organization and numbers of *nif* genes required for the synthesis and assembly of the enzyme varies greatly among these nitrogen-fixing species [6–8]. For example, in *K. oxytoca* (previously called as *K. pneumoniae*), 20 *nif* genes, *nifJHDKTYENXUSVWZMFLABQ*, organized in 7 transcriptional units are co-located within a 24 kb cluster [4], while *Paenibacillus* sp. WLY78 possesses a minimal and compact *nif* gene cluster consisting of 9 genes (*nifBnifHnifDnifKnifEnifNnifXhesAnifV*) (Fig. 1) [9, 10]. This variability in *nif* genes content is undoubtedly determined by the environmental lifestyle of each diazotroph on one hand, the minimal *nif* gene sets are probably complemented by housekeeping counterparts located elsewhere in the genome on the other hand.

Genetic and biochemical studies on the two model diazotrophs *K. oxytoca* and *A*. *vinelandii* revealed that 16 *nif* genes (*nif H*,*D*,*K*,*Y*,*T*,*E*,*N*,*X*,*U*,*S*,*V*,*Z*,*W*,*M*,*B*,*Q*) products are probably essential for efficient biosynthesis of nitrogenase [11]. It has been demonstrated that *nifH*, *nifD* and *nifK* genes encodes the structural subunits, the *nifE*, *nifN*, *nifX*, *nifB*, *nifQ*, *nifV*, *nifY* and *nifH* contribute to the synthesis and insertion of FeMo-co into nitrogenase, *nifU*, *nifS* and *nifZ* play an important role in synthesis of metalloclusters and *nifM* is required for proper folder of nitrogenase Fe protein [1–5]. However, mutations in some of these genes (notably *nifY*, *nifT*, *nifX*, *nifU*, *nifS*, *nifV*, *nifW*, *nifM* and *nifQ*) do not completely eliminate nitrogenase activity, and there is evidence that homologues elsewhere on the genome may at least partially substitute for their function [11].

Nitrogen fixation plays an important role in agriculture, and there has been a goal to engineer nitrogen fixation into cereals crops to reduce the use of chemically derived fertilizer. The complex nature of the FeMo-co assembly pathway and the large number of genes required for nitrogenase biosynthesis and maintenance of its activity represent a daunting engineering task, even in the age of systems biology. So far, the *nif* gene cluster from *K. oxytoca* and *Paenibacillus* sp. WLY78 has been successfully transferred into the prokaryotic model *E. coli* [9, 12–15]. Initially, the recombinant *E. coli* carrying a refactored *nif* cluster composed of a series of synthetic operons containing 16 *nif* genes of *K*. *oxytoca*, resulted in reduced activity (about 10 %) compared with the native system [13]. Excitingly, 57 % of wild-type activity has been recovered through modifying the synthetic *nif* genes cluster [14].

*Paenibacillus* sp. WLY78 possesses a minimal and compact *nif* gene cluster consisting of 9 genes (*nifB nifH nifD nifK nifE nifN nifX hesA nifV*) [9]. The 9 genes are organized as an operon and possess a σ^70^-dependent promoter located in front of *nifB* gene. Recently, our lab has transferred the *nif* gene operon under the control of its own native σ^70^-dependent promoter to *E. coli* JM109 [9]. The recombinant *E. coli* 78-7 synthesized catalytically active nitrogenase [8]. However, the specific activity of the enzyme expressed in *E. coli* was approximately 10 % of that observed in *Paenibacillus*. The low activity will limit the potential use of the *Paenibacillusnif* cluster in engineering nitrogen fixation into non-N_2_-fixing organisms. Thus, it is necessary to increase nitrogenase activity through synthetic biology.

In this study, two cloning and expression vectors with *Paenibacillus nif* promoter and ribosome binding site are constructed for transferring foreign genes to the recombinant *E. coli* 78-7 which carrying the *Paenibacillusnif* gene operon. A total of 28 selected genes from *Paenibacillus* and *K. oxytoca* were placed under the control of *Paenibacillus nif* promoter in these vectors and then are transferred to *E. coli* 78-7. Our results demonstrate that Fe–S cluster assembly system and electron transport system from *Paenibacillus* or *K. oxytoca* can increase *E. coli* nitrogenase activity mediated by the minimal *nif* gene cluster composed of 9 genes (*nifBHDKENXhesAnifV*). But *K. oxytoca nifWZM* and *nifQ* which are required in synthesis and maturation of nitrogenase in *K. oxytoca* can not increase any activity. Here is the first time to demonstrate that the potential electron transport genes (*pfoAB*, *fer* and *fldA*) are involved in nitrogen fixation of *Paenibacillus*. Also, it is the first time to demonstrate that *Paenibacillussuf* and *K. oxytoca nifFJ* and *nifSU* can significantly increase nitrogenase activity in *E. coli* mediated by the *Paenibacillus nif* gene operon (*nifBHDKENXhesAnifV*). Our results will provide valuable information for the incoming hot research that engineer nitrogen fixation pathway into cereal crops.

## Results

### Design of combinatorial assembly of the *nif* and *nif*-related genes

**Fig. 2:**
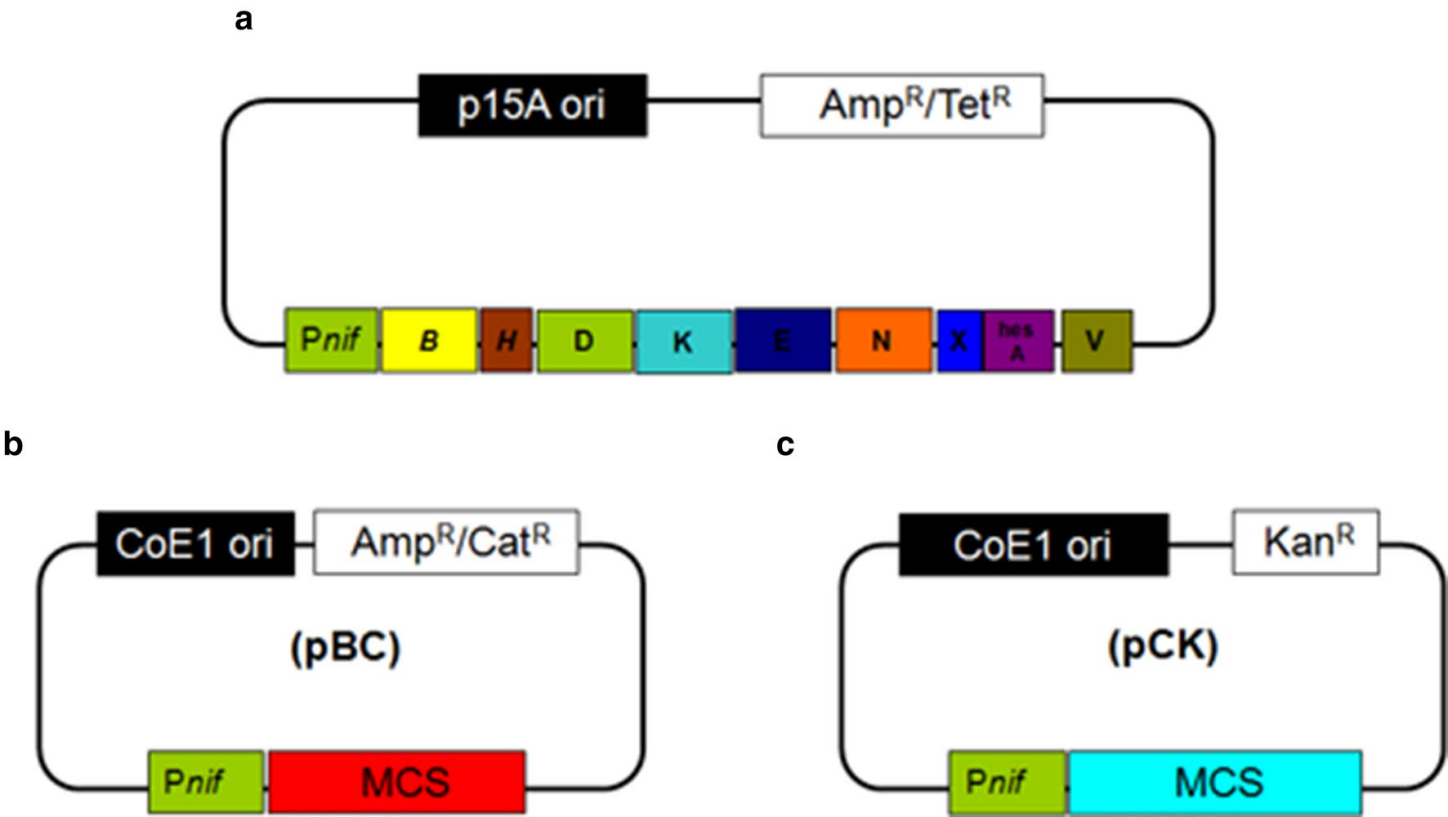
Schematic diagram outlining the structure of the pHY300PLK-derived, pBluescript II SK (+)-derived and pCAMBIA1301-derived vectors. **a** A *Paenibacillus nif* gene operon (*nifBHDKENXhesAnifV*) carried in vector pHY300PLK. **b**, **c** Vectors constructed in this study. MCS, multiple clone sites

*E. coli* 78-7 is a recombinant strain carrying a *Paenibacillus nif* gene operon (*nifBHDKENXhesAnifV*) in vector pHY300PLK [9, 16] (Fig. 2a). As described in methods, two vectors carrying *Paenibacillusnif* promoter, ribosome-binding site and the multiple cloning site (MCS) are constructed (Fig. 2b, c) for expressing other *nif* or *nif*-related genes in *E. coli* 78-7. The two vectors can coexist in *E. coli* cells with plasmid pHY300PLK. Then the two vectors carrying foreign genes from *Paenibacillus* sp. WLY78 or *K. oxytoca* were separately or combinationally transferred to *E. coli* 78-7 (Additional file 1: Table S1, Additional file 2: Table S2).

### *Paenibacillus* Suf system can increase nitrogenase activity of the recombinant *E. coli* 78-7

Nitrogenase is a complex [Fe–S] enzyme. Many diazotrophs, such as *K. oxytoca* and *A. vinelandii*, contain *nifU* and *nifS* whose products were involved in the assembly of [Fe–S] clusters of nitrogenase [2–5]. NifU and NifS separately provide the Fe and S required for nitrogenase maturation. The genome of *Paenibacillus* sp. WLY78 does not have *nifSU*, but contains iron-sulfur cluster assembly systems: a complete *suf* (*sufCBSUD)* operon and a partial *isc* system (*iscSR*). Similarly, there are no *nifS* and *nifU* in *E. coli*, but *E. coli* has two iron-sulfur cluster assembly systems: the *sufABCDSE* operon and the *isc* system composed of *iscR*, *iscS*, *iscU*, *iscA*, *hscB*, *hscA*, *fdx*, and *orf3* [17]. The *nif* gene operon from *Paenibacillus* sp. WLY78 could enable *E. coli* to fix nitrogen, suggesting that the assembly of Fe–S clusters for the nitrogenase was provided by *E. coli* iron-sulfur cluster assembly systems.

**Fig. 3:**
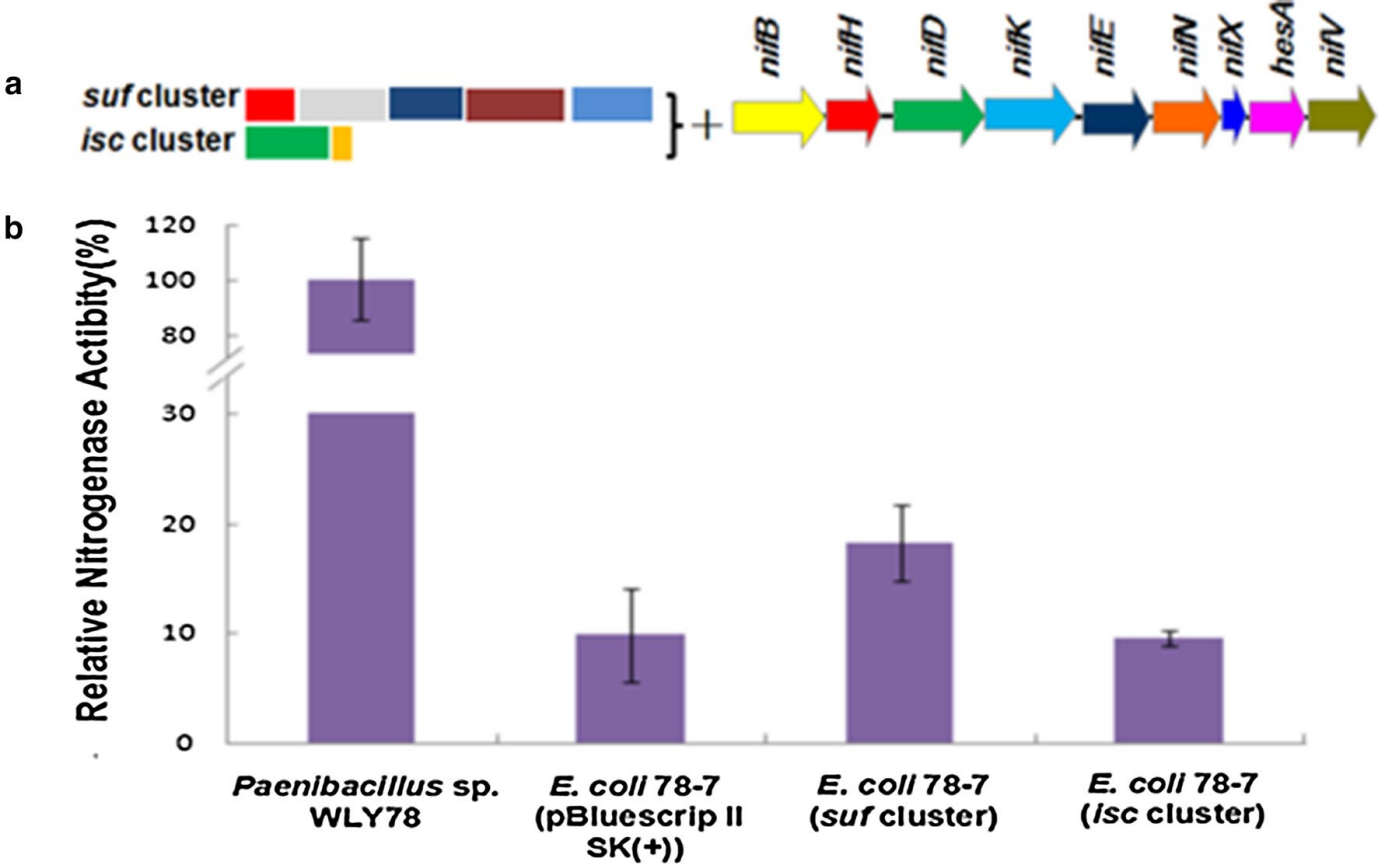
Assembly and functional analysis of the *K. oxytoca* Fe–S cluster assembly system (*nifUS*) in *E. coli* 78-7. **a** Linear view of the Fe–S cluster assembly gene region in pBluescriptII SK (+)-derived plasmid. **b** Relative nitrogenase activity of wild-type *Paenibacillus* sp. WLY78, *E. coli* 78-7 [pBluescriptIISK (+)] and *E. coli* 78-7 (*nifUS*). *E. coli* 78-7 [pBluescriptII SK (+)] was used as a control. Each experiment was repeated at least three times, and the *error bars* represent standard error

In this study, the *suf* (*sufCBSUD)* operon and *iscSR* system from *Paenibacillus* sp. WLY78 are placed under the control of *Paenibacillus nif* promoter, respectively, and then are separately transferred into the recombinant *E. coli* 78-7. As shown in Fig. 3, the *suf* (*sufCBSUD)* operon can increase nitrogenase activity of *E. coli* 78-7 from 10 to 20 %, while *iscSR* system cannot increase any activity. The data suggest that the *suf* (*sufCBSUD)* operon plays an important role in Fe–S cluster assembly in nitrogenase synthesis of *Paenibacillus*.

### *Klebsiella oxytoca nifSU* can increase nitrogenase activity of the recombinant *E. coli* 78-7

**Fig. 4:**
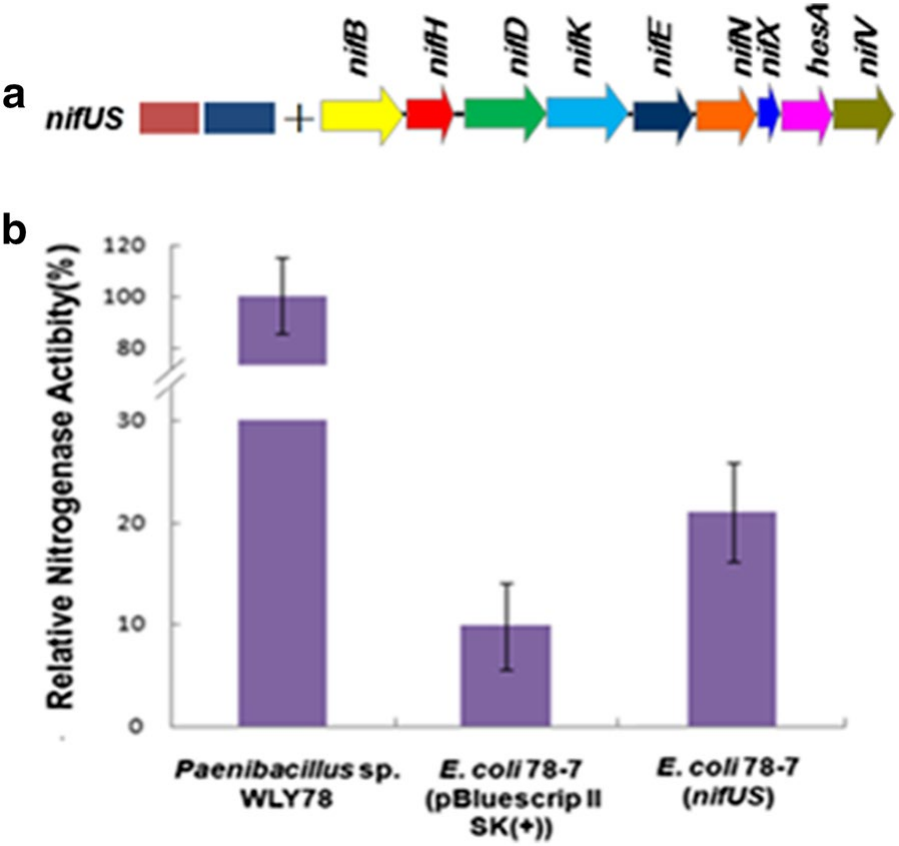
Assembly and functional analysis of the *Paenibacillus* Fe–S cluster assembly systems (*suf* and *isc*) in *E. coli*. **a** Linear view of the Fe–S cluster assembly gene region in pBluescriptII SK (+)-derived plasmid. **b** Relative nitrogenase activity of wild-type *Paenibacillus* sp. WLY78, *E. coli* 78-7 [pBluescriptII SK (+)], *E. coli* 78-7 (*suf*) and *E. coli* 78-7 (*isc*). *E. coli* 78-7 [pBluescriptII SK (+)] was used as a control. Each experiment was repeated at least three times, and the *error bars* represent standard error

As described in methods, *K. oxytoca nifSU* gene cluster was placed under the control of *Paenibacillus nif* promoter and then was transferred to *E. coli* 78-7. As shown in Fig. 4, *K. oxytoca nifSU* can increase activity of *E. coli* 78-7 from 10 to 19.5 %. Our data that *K. oxytoca nifSU* or *Paenibacillus sufCBSUD* can increase nitrogenase activity of *E. coli* 78-7 is consistent with the fact that nitrogenase is a complex [Fe–S] enzyme which contains 38 Fe atoms, 40 S atoms and 2 Mo atoms.

### The potential electron transporters from *Paenibacillus* can increase nitrogenase activity of the recombinant *E. coli* 78-7

Nitrogen fixation is carried out by the enzyme nitrogenase, which transfers electrons originating from low-potential electron carriers, such as flavodoxin or ferredoxin molecules, to molecular N_2_ [18]. In *K. oxytoca*, the physiological electron flow to nitrogenase involves specifically the products of the *nifF* and *nifJ* genes [19]. The *nifF* gene product, a flavodoxin, mediates electron transfer from the *nifJ* gene product, a pyruvate: flavodoxin oxidoreductase, to the Fe protein of nitrogenase [20–23].

Unlike *K. oxytoc*a *nif* gene cluster, *Paenibacillus nif* gene cluster dose not have *nifF* and *nifJ*. Genome sequence analysis revealed that there are several genes encoding ferredoxin, flavodoxin and flavodoxin oxidoreductase in the genome of *Paenibacillus* sp. WLY78. For example, *fer* and *COG3411* encode ferredoxin, *fldA* and *fldB* encode flavodoxin, and *fpr* encodes ferredoxin-NADP reductase, *nfrA* encodes NAD(P)H-flavin oxidoreductase, and *pfoAB* separately encode pyruvate: ferredoxin oxidoreductase gamma subunit and alpha subunit. Of these genes, *fldA* and *fldB* shows 30 % identity with *K. oxytoca nifF*, and *pfoAB* exhibit 33 % identity with *K. oxytoca nifJ*, but other genes do not show identity with *K. oxytoca nifF or nifJ.*

**Fig. 5:**
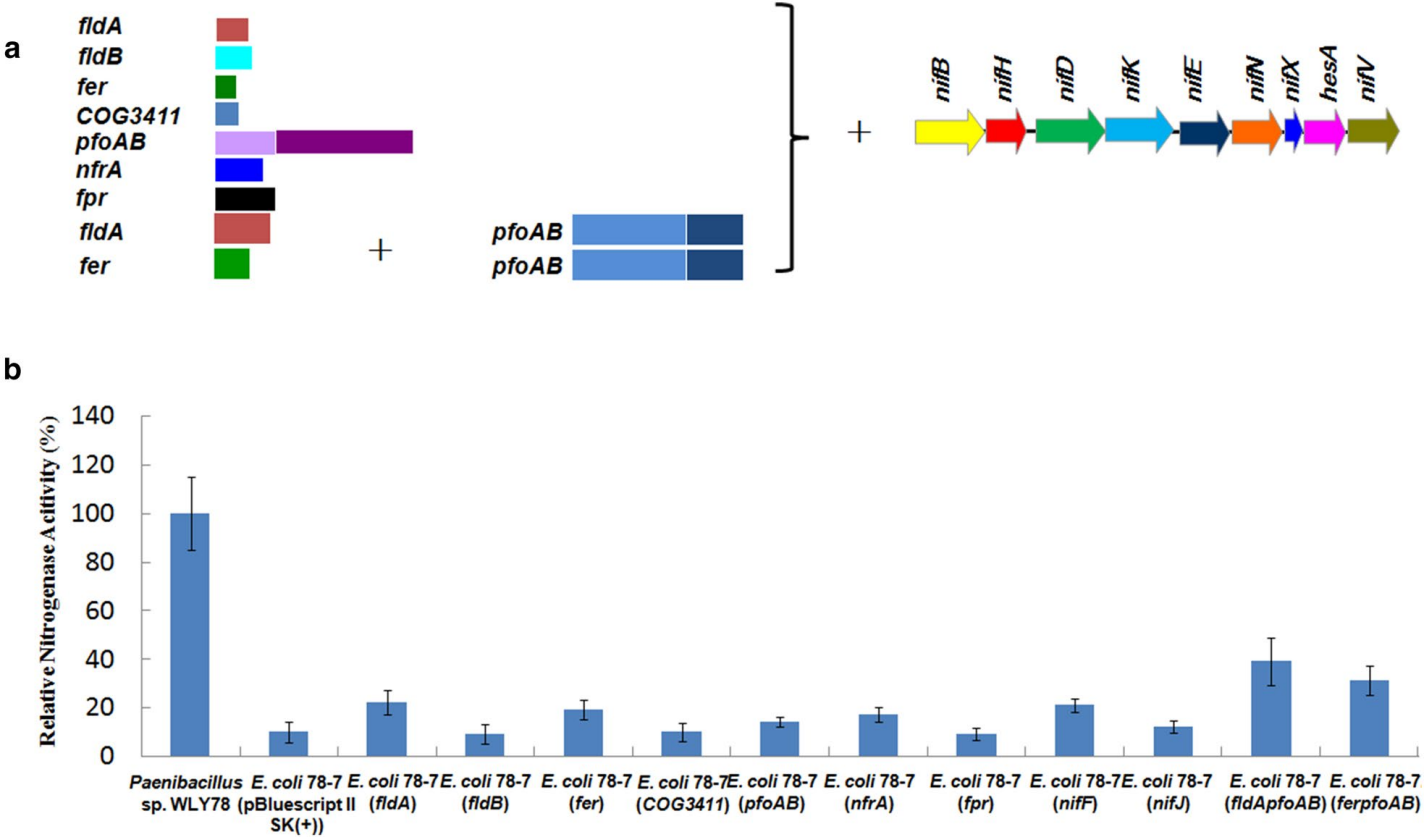
Assembly and functional analysis of the potential *Paenibacillus* electron transport genes in *E. coli* 78-7. **a** Linear view of the potential *Paenibacillus* electron transport genes. **b** Relative nitrogenase activity of wild-type *Paenibacillus* sp. WLY78, *E. coli* 78-7 [pBluescriptII SK (+)] and *E. coli* 78-7 strains carrying *Paenbacillus* genes. *E. coli* 78-7 [pBluescriptII SK (+)] was used as a control. Each experiment was repeated at least three times, and the *error bars* represent standard error

Furthermore, the *fer*, *fldA*, *fldB* and *COG3411*, the orthlogs of *K. oxytoca nifF*, were separately transferred into the recombinant *E. coli* 78-7. As shown in Fig. 5, each of *fer* and *fldA* can increase nitrogenase of *E. coli* 78-7 from 10 to 20.1 %, while *fldB* and *COG3411* cannot increase any activity. The data suggest that *fer* (ferredoxin) and *fldA* (flavodoxin) might be an electron transporter of nitrogenase. Our data are consistent with the previous report that either flavodoxins or ferredoxins are the direct electron donor to nitrogenase in diazotrophic bacteria [21].

Furthermore, *nfrA*, *fpr* and *pfoAB*, the orthlogs of *K. oxytoca nifJ*, were separately transferred into the recombinant *E. coli* 78-7. As shown in Fig. 5, *pfoAB* increase nitrogenase activity from 10 to 15 %, while *nfrA* and *fpr* do not increase any activity. The data suggest that *pfoAB* play a role in nitrogen fixation. Notably, the nitrogenase activity of *E. coli* 78-7 is increased to 35.1 and 40.1 %, respectively, when *pfoAB* is combined with *Paenibacillus fer* gene (*Paenibacillus*-*ferproAB*) or *PaenibacillusfldA* gene (*Paenibacillus*-*fldAproAB*). We deduce that in *Paenibacillus*, *pfoAB* (pyruvate: ferredoxin oxidoreductase) might be involved in the pyruvate breakdown to yield electrons, and then *fldA* and *fer* mediate electron to nitrogenase.

### *Klebsiella oxytoca nifF* and *nifJ* can increase nitrogenase activity of the recombinant *E. coli* 78-7

**Fig. 6:**
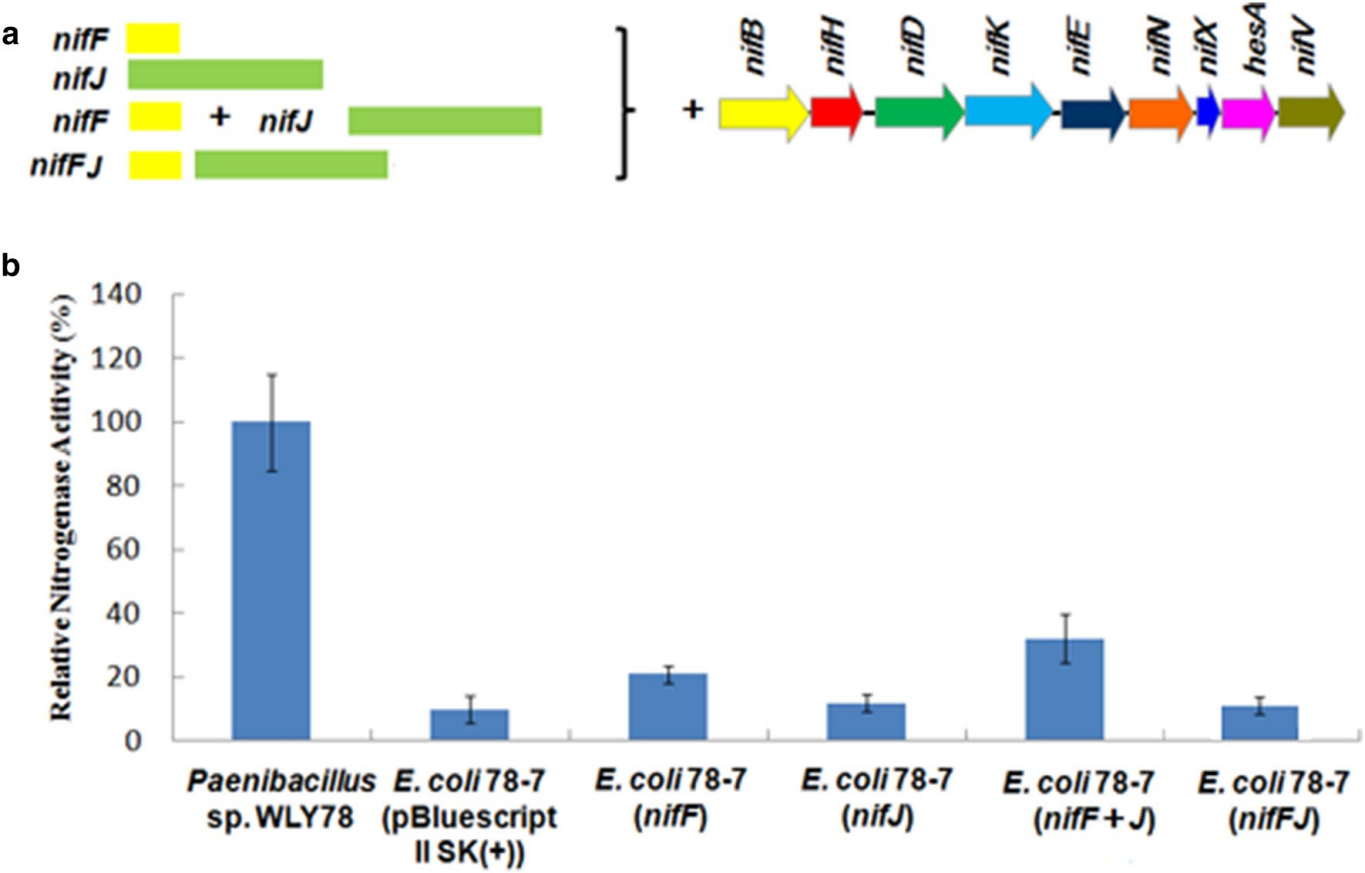
Assembly and functional analysis of the *K. oxytoca* electron transport genes in *E. coli* 78-7. **a** Linear view of the *K. oxytoca* electron transport genes. **b** Relative nitrogenase activity of wild-type *Paenibacillus* sp. WLY78, *E. coli* 78-7 [pBluescriptII SK (+)] and *E. coli* 78-7 strains carrying *K. oxytoca* genes. *E. coli* 78-7 [pBluescriptII SK (+)] was used as a control. Each experiment was repeated at least three times, and the *error bars* represent standard error

As shown in Fig. 6, *K. oxytocanifF* and *nifJ*, whose products are electron transporters, can increase nitrogenase of *E. coli* 78-7 from 10 to 20.4 and 12,1 %, respectively. When *nifF* and *nifJ* were carried in two different vectors and co-transferred into *E. coli* 78-7, the activity was increased from 10 to 32 %. However, nitrogenase activity could not be increased when *nifF* and *nifJ* were assembled as an operon. Our results are consistent with the report that coordinated and balanced expression of *nifF* and *nifJ* genes is important for nitrogenase activity in *E. coli* carrying *K. oxytoca nif* clusters [15].

### Combination of Fe–S cluster synthesis system and electron transporters can significantly increase nitrogenase activity

**Fig. 7:**
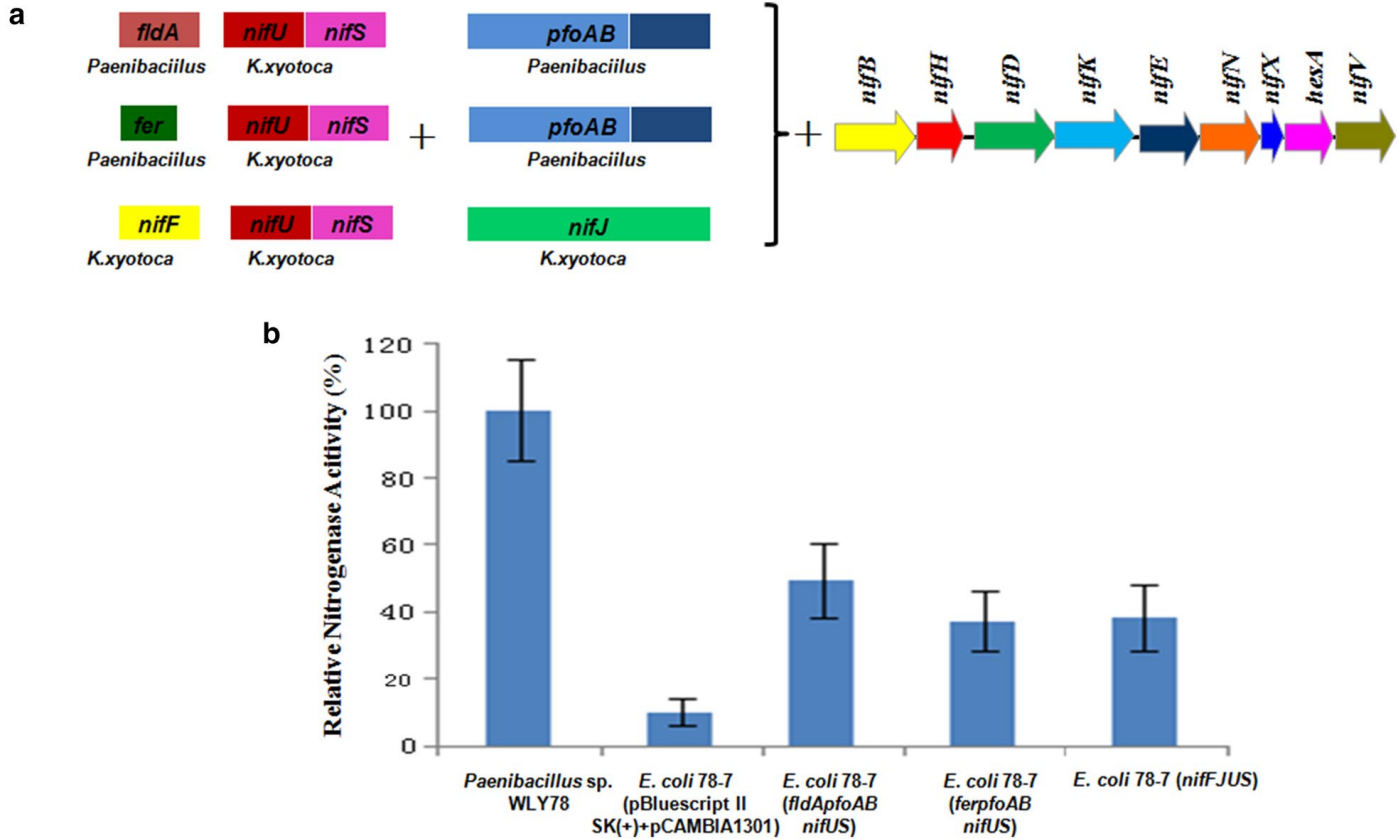
Assembly of Fe–S cluster synthesis system and electron transporters in *E. coli* 78-7. **a** Linear view of the genes. **b** Relative nitrogenase activity of wild-type *Paenibacillus* sp. WLY78, *E. coli* 78-7 [pBluescriptII SK (+) and pCAMBIA1301] and *E. coli* 78-7 strains carrying different genes. *E. coli* 78-7. [pBluescriptII SK (+) and pCAMBIA1301] was used as a control. Each experiment was repeated at least three times, and the *error bars* represent standard error

As described above, the (potential) electron transporters and iron-sulfur cluster assembly systems from *Paenibacillus* sp. WLY78 or *K. oxytoca* can increase activity of *E. coli* 78-7. Here, combination of the (potential) electron transporters and iron-sulfur cluster assembly system was transferred to *E. coli* 78-7. Considering that *K. oxytoca nifSU* genes are much shorter and easier to operate in gene cloning than *Paenibacillus suf* system, *K. oxytoca nifSU* genes were used in this combined assembly with the (potential) electron transporters from *Paenibacillus* sp. WLY78 or *K. oxytoca.* As shown in Fig. 7, the combined Kp-*nifJnifFnifUS*, WLY78-*ferpfoAB*- Kp-*nifUS* and WLY78-*fldApfoAB* -Kp-*nifUS* increase activity from 10 to 39.1, 45.1 and 50.1 %, respectively. The highest activity obtained by WLY78-*fldApfoAB* -Kp-*nifUS* suggests that *fldApfoAB* are the electron transport of nitrogenase in *Paenibacillus.*

### *Klebsiella oxytoca nifWZM* and *nifQ* can not increase nitrogenase activity

It was reported that the *nifW* and *nifZ* genes seem to be involved in MoFe protein maturation, while *nifM* is required for proper folding of nitrogenase Fe protein [1, 3]. *nifM* mutants of *K.oxytoca* and *A. vinelandii* were unable to synthesise action Fe protein [24–26].Unlike in *K. oxytoca*, *Paenibacillus* has not the *nifWZM* genes. And *E. coli* has also not the *nifWZM* genes. In this study, the *K. oxytocanifWZM* genes were transferred to *E. coli* 78-7, but the nitrogenase activity was not enhanced by these genes. The data suggest that the requirement of *nifWZM* genes on maturation of nitrogenase vary greatly among diazotrophs.

**Fig. 8:**
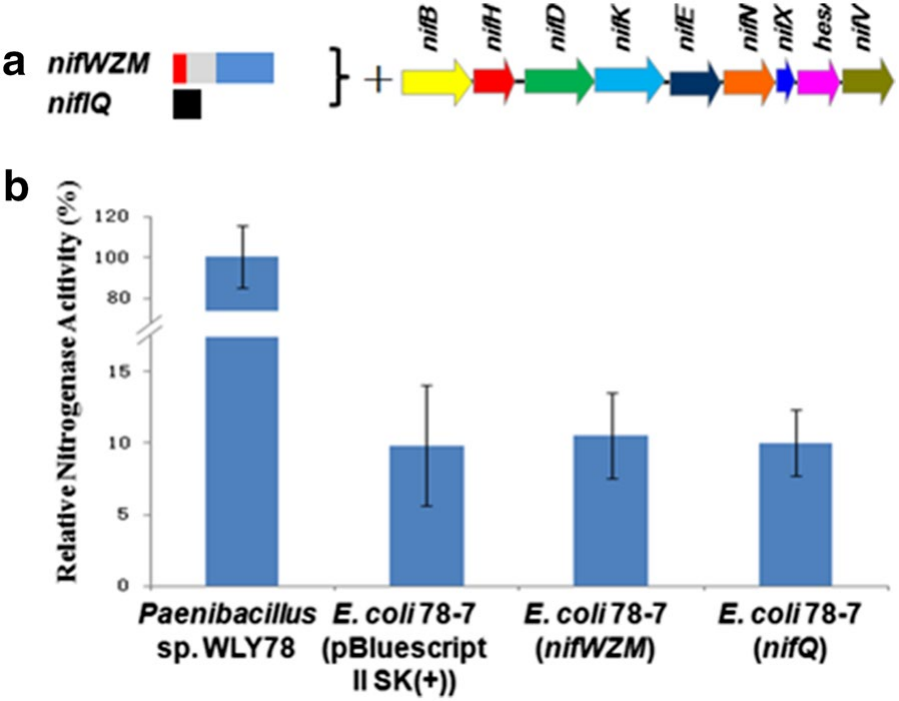
Assembly and functional analysis of the *K. oxytoca* *nifWZM* and *nifQ* in *E. coli* 78-7. **a** Linear view of the *nifWZM* and *nifQ* genes region in pBluescriptII SK (+)-derived plasmid. **b** Relative nitrogenase activity of wild-type *Paenibacillus* sp. WLY78, *E. coli* 78-7 [pBluescriptII SK (+)], *E. coli* 78-7 (*nifWZM*) and *E. coli* 78-7 (*nifQ*). *E. coli* 78-7 [pBluescriptII SK (+)] was used as a control. Each experiment was repeated at least three times, and the *error bars* represent standard error

NifQ has been implicated in the processing of molybdenum specifically for the biosynthesis of FeMo-co [1, 2]. Unlike in *K. oxytoca*, *Paenibacillus* dose not have *nifQ* gene. In this study, the *K. oxytocanifQ* gene was transferred to *E. coli* 78-7, but the nitrogenase activity was not enhanced by *nifQ* gene. The result indicates that *K. oxytocanifQ* is not involved in the processing of molybdenum specifically for the biosynthesis of FeMo-co of nitrogenase encoded by *Paenibacillus nif* genes (Fig. 8).

## Discussion

Our recent studies have revealed that the genome of *Paenibacillus* sp. WLY78 contains a minimal *nif* gene cluster composed of nine genes *nifBHDKENXhesAnifV* and the *nif* operon under the control of its own *nif* promoter enabled *E. coli* to synthesize the active nitrogenase [9]. However, the specific activity of the enzyme expressed in *E. coli* was approximately 10 % of that observed in *Paenibacillus*. In this study, synthetic biology was used to determine whether 28 selected genes from *Paenibacillus* sp. WLY78 and *K. oxytoca* can increase nitrogenase activity of the recombinant *E. coli* 78-7.

Compared with *K. oxytoca nif* clusters, one of the notable absences in the minimal *Paenibacillusnif* gene cluster is the two genes *nifS* and *nifU*, which provide the nitrogen fixation-specific iron-sulfur cluster assembly. The genome of *Paenibacillus* sp. WLY78 does not have *nifSU*, but contains iron-sulfur cluster assembly systems: a complete *suf* (*sufCBSUD)* operon and a partial *isc* system (*iscSR*). In this study, we demonstrate that *Paenibacillussuf* (*sufCBSUD)* operon can increase the nitrogenase activity of the recombinant *E. coli* 78-7, and *K.oxytoca nifSU* also can increase activity. The results reveal that iron-sulfur cluster assembly system specific for Fe–S cluster of nitrogenase is very important to nitrogen fixation. The results also imply that although *E. coli* iron-sulfur cluster assembly system can support the synthesis of active nitrogenase, it cannot fully support the requirement for synthesis of Fe–S cluster.

It was reported that pyruvate is a major source of electrons in diazotrophic *Clostridium pasteurianum* and *Bacillus polymyxa* (now called as *Paenibacillus polymyxa*) [22]. In *K. oxytoc*a, the pyruvate oxidoreductase (*nifJ* gene product) was responsible for the pyruvate breakdown to yield electrons, and then the flavodoxin (the *nifF* gene product) mediates electron transfer to the Fe protein of nitrogenase [23].The *Paenibacillus* sp. WLY78 *nif* gene operon does not contain homologs of *nifF* (encoding a flavodoxin) and *nifJ* (pyruvate: flavodoxin oxidoreductase) which provide the electron transport chain to nitrogenase in some diazotrophs [22, 23]. In this study, we search and find that the *fer* (ferredoxin), *COG3411* (ferredoxin), *fldA (*flavodoxin), *fldB* (flavodoxin), *fpr (*ferredoxin-NADP reductase), *nfrA (*NAD(P)H-flavin oxidoreductase) and *pfoAB* (pyruvate: ferredoxin oxidoreductase) are scared on *Paenibacillus* genomic regions outside of *nif* genes cluster. When each of these genes is separately transferred to *E.coli* 78-7, only *fer*, *fldA* and *pfoAB* can increase activity. Combinational assembly of *fer* or *fldA* with *pfoAB* can significantly increase activity. We deduce that *pfoAB* gene product (pyruvate: ferredoxin oxidoreductase) might be involved in the pyruvate breakdown to yield electrons, and then *fldA* and *fer* mediate electron to nitrogenase. Here is the first time to reveal that *pfoAB*, *fer* and *fldA* genes are involved in nitrogen fixation mediated by *Paenibacillus nif* genes. Notably, PfoAB shows 33 % identity with *K. oxytoca* NifJ. But the *K. oxytoca nifJ* gene product is a single subunit, while *Paenibacillus pfoAB* gene products are two subunits. Here, we show that both *fldA* and *fer* can enhance nitrogenase activity, suggesting that the both genes can transfer electron to Fe protein*. fldA* also exists in *E. coli* and *K. oxytoca* [27]. Whether *fldA* is involved in transferring electron to Fe protein of nitrogenase in *K. oxytoca* is not known. In *E. coli*, FldA and Fpr (the NADPH-dependent flavin adenine dinucleotide (FAD) containing flavodoxin/ferredoxin reductase) are required for the activation of key enzymes in the synthesis of methionine, biotin, pyruvate and deoxyribonucleotides [28–30]. Remarkably, the Fpr-FldA redox system can effectively deliver electrons to non-physiological partners, which include a variety of P450 enzymes [31]. Thus, we deduce that the Fpr-FldA redox system might be responsible for electron transport to nitrogenase in *E. coli.*

Also, we demonstrate that each of *K. oxytoca nifJ* and *nifF* genes can increase nitrogenase activity of *E. coli* 78-7. The higher activity is obtained when *K. oxytoca nifJ* and *nifF* genes were carried in different vectors. However, nitrogenase could not be increased when *nifF* and *nifJ* were assembled as an operon. Our results are consistent with the report that coordinated and balanced expression of *nifF* and *nifJ* genes is important for nitrogenase activity in *E. coli* carrying *K. oxytoca nif* clusters.

Furthermore, in order to increase nitrogenase activity of *E. coli* 78-7, we design to assemble electron transport genes from *Paenibacillus* or *K. oxytoca* with Fe–S cluster synthesis genes from *Paenibacillus* or *K. oxytoca*. Considering *K. oxytocanifSU* are much shorter and easier to operate in gene cloning than *Paenibacillus suf* operon, *K. oxytocanifSU* are used in the combinational assembly with electron transport genes. The combinational assembly of *Paenibacillus fer*-*pfoAB* with *K. oxytocanifSU*, *PaenibacillusfldA*-*pfoAB* with *K. oxytocanifSU*, *K. oxytocanifF* and *nifJ* with *nifSU* was constructed. Our results demonstrated that these combinational assemblies can significantly increase activity. Especially, *Paenibacillus fldA*-*pfoAB* with *K. oxytocanifSU* can recover 50 % activity of wild-type *Paenibacillus*. Our results provide valuable information for engineering nitrogen fixation pathway into cereal crops.

The *nifW* and *nifZ* genes seem to be involved in MoFe protein maturation [1, 3], while *nifM* is required for proper folding of nitrogenase Fe protein in *K. oxytoca* [24–26]. The *nifWZM* genes are not only absent in the *Paenibacillus nif* cluster, but also in the *nif* clusters of the Gram-positive *Clostridium*, *Heliobacterium chlorum* and archaeal *Methanococcus maripaludis* [32]. Also, *nifM* is absent in *Rhizobia*, such as *Azorhizobium caulinodans*, *Sinorhizobium meliloti* and *Rhizobium leguminosarum* [7]. Our current studies demonstrate that *K. oxytocanifWZM* can not increase nitrogenase activity of *E. coli* 78-7.These data support that the *nifWZM* genes are not required for nitrogen fixation in *Paenibacillus* sp. WLY78. Whether the functions of the *nifWZM* genes are replaced by other components scared in the genome of *Paenibacillus* sp. WLY78 and *E. coli* is not known.

It was reported that *nifM* encodes a cis–trans peptidyl prolyl isomerase and are involved in proper folding of nitrogenase Fe protein in *Azotobacter vinelandii* [26]. When the conserved Pro258 located in the C-terminal region of Fe protein (NifH) of *A. vinelandii*, which wraps around the other subunit in the NifH dimer, is replaced by serine, the correct folding of Fe protein (NifH) can acquire NifM independence [26, 33]. We compare the *Paenibacillus* NifH sequence with other NifH sequences and find that *Paenibacillus* contains the conserved proline residues identified in other NifH sequences that are considered to be potential substrates for NifM (Additional file 3: Figure S1). It is possible that other amino acid substitutions in NifH may enable assembly of Fe protein in the absence of NifM.

It has been demonstrated that *nifQ* is required for nitrogen fixation in *K. oxytoca* and *A. vinelandii*. Recent results show that NifQ is an iron-sulfur protein with a redox-responsive [Fe–S] cluster and NifQ is also a molybdoprotein that serves as a direct molybdenum donor for FeMo-co synthesis, replacing molybdate in the in vitro FeMo-co synthesis assay [34]. Electron paramagnetic resonance (EPR) spectroscopic studies indicated that NifQ carries a [Mo-Fe3-S4] cluster, and that the presence of this metal cluster in NifQ correlates with its ability to support in vitro FeMo-co synthesis [1]. However, there is no *nifQ* in diazotrophic *Paenibacillus*, *Clostridium*, *cyanobacteria* and *Frankia* [35, 36]. This study demonstrates that *K. oxytoca nifQ* did not enhance the activity of *E. coli* 78-7. Interestingly, there is a *hesA* gene located within the *nif* clusters of diazotrophic *Paenibacillus*, *cyanobacteria* and *Frankia* [35, 36]. Our deletion analysis demonstrates that *hesA* is important for nitrogenase activity, but the function of *hesA* in nitrogen fixation has not so far been determined. HesA belongs to the ThiF-MoeB-HesA family which engages in an ATP-dependent process that activates the C-terminus of partner ubiquitin-like proteins by forming an acyladenylate complex that facilitates sulfur transfer [37, 38]. It is to speculate that HesA may perform a role in metallocluster biosynthesis. These data suggest that synthesis and maturation of nitrogenase exhibit some different features between *nif* gene clusters of Gram-negative *K. oxytoca/A. vinelandii* and Gram-positive *Paenibacillus*.

## Conclusion

A total of 28 selected genes from *Paenibacillus* sp. WLY78 and *K. oxytoca* are separately or combinationally transferred into the recombinant *E. coli* 78-7. Of these 28 genes, 8 genes (*pfoAB*, *fldA*, *fldB*, *fer*, *fpr*, *nfrA* and COG3411) encoding the potential electron transport and 2 gene clusters (*suf* and *isc*) encoding Fe–S cluster synthesis are from *Paenibacillus* sp. WLY78, and 8 genes (*nifF*, *nifJ*, *nifSU*, *nifWZM* and *nifQ*) specific for electron transport, Fe–S cluster synthesis and maturation of nitrogenase are from *K. oxytoca*. Our results demonstrate that *Paenibacillussuf* operon and the potential electron transporter genes (*pfoAB*, *fldA* and *fer*) can increase nitrogenase activity. Also, *K. oxytocanifSU* and *nifFJ* can increase nitrogenase activity. Especially, combined assembly of the potential electron transporter genes (*pfoABfldA*) with *K. oxytoca nifSU* recovers 50.1 % of wild-type activity. Also, we demonstrate that *nifWZM* and *nifQ* can not increase activity, suggest ing that the requirement of *nifWZM* and *nifQ* genes on maturation of nitrogenase vary greatly among diazotrophs. This study will provide valuable insights for the enhancement of nitrogenase activity in heterogeneous host and will provide guidance for engineering cereal plants with minimal *nif* genes.

## Methods

### Strains and medium

*Paenibacillus* sp. WLY78, a nitrogen-fixer, was isolated by our lab [9]. The recombinant *E. coli* 78-7 which carries an 11 kb *nif* genes cluster from *Paenibacillus* sp. WLY78 was constructed by our lab [9]. *Paenibacillus* sp. WLY78 and *E. coli* strains were routinely grown in LB or LD medium (per liter contains: 2.5 g NaCl, 5 g yeast and 10 g tryptone) at 30℃ with shaking. When appropriate, antibiotics were added in the following concentrations: 50 m g/ml kanamycine, 100 m g/ml ampiciline, and 12.5 m g/ml tetracycline for maintenance of plasmids. Nitrogen-free and nitrogen-deficient media were used for assay of nitrogenase activity. Nitrogen-free medium contained (per liter) 10.4 g Na_2_HPO_4_, 3.4 g KH_2_PO_4_, 26 mg CaCl_2_·2H_2_O, 30 mg MgSO_4_, 0.3 mg MnSO_4_, 36 mg Ferric citrate, 7.6 mg Na_2_MoO_4_·2H_2_O, 10 μg *p*-aminobenzoic acid, 5 μg biotin and 4 g glucose as carbon source. Nitrogen-deficient medium contained 2 mM glutamate as nitrogen source in nitrogen-free medium [9].

### Nitrogenase activity assays

For nitrogenase activity assays, *Paenibacillus* sp.WLY78 and the recombinant *E. coli* strains were grown in 5 ml of LD media (supplemented with antibiotics) in 50 ml flasks shaken at 250 rpm for 16 h at 30 °C. The cultures were collected by centrifugation, washed three times with sterilized water and then resuspended in nitrogen-deficient medium containing 2 mM glutamate as nitrogen source (supplemented with antibiotics for the engineered *E. coli* strains when necessary) to a final OD600 of 0.2–0.4. Then, 1 ml of the culture was transferred to a 25-ml test tube and the test tube was sealed with robber stopper. The headspace in the tube was then evacuated and replaced with argon gas [14]. After incubating the cultures for 6–8 h at 30℃ with shaking at 250 rpm, C_2_H_2_ (10 % of the headspace volume) was injected into the test tubes. After incubating the cultures for a further 3 h, 100 ml of culture headspace was withdrawn through the rubber stopper with a gas tight syringe and manually injected into a HP6890 gas chromatograph to quantify ethylene production. All treatments were in three replicates and all the experiments were repeated three or more times.

### Construction of cloning and expression vectors

Since *E. coli* 78-7 carries a *Paenibacillus nif* gene operon in vector pHY300PLK which is a shuttle vector with two replication origins:one is p15A which can be reproduced in *E. coli* and the other is a pAMα1 replicon from a plasmid pAMα1 of *Streptococcus faecalis* which can be reproduced in Gram-positive *Bacillus* [16]. The p15A replicon allows itself to coexist in *E. coli* cells with plasmids of the ColE1 compatibility group (e.g., pBR322, pUC19, pBluescript II SK (+)). Thus, two cloning and expression vectors carrying a *Paenibacillusnif* promoter and a ribosome binding site are here constructed in order to express foreign genes in the recombinant *E.coli* 78-7. The first vector (here called pBC) contains the backbone derived from pBluescript II SK (+), including the *E. coli* origin ColE1, ampiciline resistance marker amp and the multiple cloning sites (MCS). A 307 bp *Paenibacillus nif* promoter region (carrying XhoI and HindIII restriction sites at both ends) from the genomic DNA of *Paenibacillus* sp. WLY78 and a 1.2 kb chloramphenicol resistance gene fragment (carrying KpnI restriction sites at both ends) from the plasmid pPR9TT were PCR amplified and then ligated to the ampicillin-resistant plasmid pBluescript II SK (+), resulting vector pBC. The second vector (here named as pCK) contains the *E. coli* origin ColE1, kanamycine resistance marker kan and the multiple cloning site (MCS) from plasmid pCAMBIA1301 and a 307 bp *nif* promoter region (carrying XhoI and HindIII restriction sites at both ends) from the genomic DNA of *Paenibacillus* sp. WLY78.

### Construction of recombinant plasmids and recombinant *E. coli* strains

Here, a total of fourteen DNA fragments including 28 genes were PCR amplified from *Paenibacillus* sp. WLY78 and *K. oxytoca*. First, nine DNA fragments (488 bp, 246 bp, 899 bp, 553 bp, 345 bp, 827 bp, 1045 bp, 3306 bp, 6455 bp and 1345 bp) which contain *fldA*, *fer*, *fldB*, *COG3411*, *nfrA*, *fpr*, *pfoAB* genes, *suf* and *isc* genes cluster of *Paenibacillus* sp. WLY78, respectively, were PCR amplified. Five DNA fragments (564, 3543, 2090, 1534 and 533 bp) containing *nifF*, *nifJ*, *nifUS*, *nifWZM* and *nifQ* genes, respectively, were PCR amplified from *K. oxytoca* M5a1. The *fldA*, *fer*, *fldB*, *COG3411*, *nifF*, *nifWZM* and *nifQ* gene fragments carried BamHI and XbaI target sites flanking the coding region. The *nfrA*, *fpr*, *pfoAB* and *nifJ* gene fragments carried HindIII and BamHI target sites flanking the coding region. The *suf* and *isc* cluster and *nifUS* genes fragment carried XbaI and SacI target sites at both ends. Each of these gene or genes cluster was cloned to the plasmid pBC and was placed under the control of *nif* promoter. The *nifF*, *fldA*, *fer*, *nifUS* genes were cloned into the vector pCK, respectively. Primers for PCR, recombinant plasmids and strains are listed in Additional file 1: Tables S1, Additional file 2: Table S2, Additional file 4: Table S3 and Additional file 5: Table S4.

## Additional files

10.1186/s12934-016-0442-6 The vectors constructed in this study.
10.1186/s12934-016-0442-6 Bacterial strains and plasmids used in this study.
10.1186/s12934-016-0442-6 Comparison of different NifH proteins showing the Proline258 is conserved in *Paenibacillus*.
10.1186/s12934-016-0442-6 The recombinant strains constructed in this study.
10.1186/s12934-016-0442-6 Primers used in this study.
