# Supplementary material for: Using synthetic biology to increase nitrogenase activity
Source: Microb Cell Fact. 2016 Feb 20;15:43. doi: 10.1186/s12934-016-0442-6 (PMC4761190; doi:10.1186/s12934-016-0442-6)
Supplement: Supplementary file 1 — 10.1186/s12934-016-0442-6 The vectors constructed in this study. [file 12934_2016_442_MOESM1_ESM.pdf]

Table S1. The vectors constructed in this study.

Vector	Promoter	Foreign genes
pBC (pBluescriptⅡ derative)	*nif*	None genes
pBC-J	*nif*	*K. oxytoca nifJ*
pBC-F	*nif*	*K. oxytoca nifF*
pBC-US	*nif*	*K. oxytoca nifUS*
pBC-WZM	*nif*	*K. oxytoca nifWZM*
pBC-Q	*nif*	*K. oxytoca nifQ*
pBC-fldA	*nif*	*Paenibacillus fldA*
pBC-fer	*nif*	*Paenibacillus fer*
pBC-fldB	*nif*	*Paenibacillus fldB*
pBC-COG3411	*nif*	*Paenibacillus COG3411*
pBC-pfoAB	*nif*	*Paenibacillus pfoAB*
pBC-nfrA	*nif*	*Paenibacillus nfrA*
pBC-fpr	*nif*	*Paenibacillus fpr*
pBC-suf	*nif*	*Paenibacillus suf cluster*
pBC-isc	*nif*	*Paenibacillus isc cluster*
pCK (pCAMBIA1301 derative)	*nif*	None genes
pCK-nifF	*nif*	*K. oxytoca nifF*
pCK-nifFUS	*nif*	*K. oxytoca nifF* and *nifUS*
pCK-fldA	*nif*	*Paenibacillus fldA*
pCK-fer	*nif*	*Paenibacillus fer*
pCK-fldAUS	*nif*	*Paenibacillus fldA* and *K. oxytoca nifUS*
pCK-ferUS	*nif*	*Paenibacillus fer* and *K. oxytoca nifUS*
