# Supplementary material for: Using synthetic biology to increase nitrogenase activity
Source: Microb Cell Fact. 2016 Feb 20;15:43. doi: 10.1186/s12934-016-0442-6 (PMC4761190; doi:10.1186/s12934-016-0442-6)
Supplement: Supplementary file 2 — 10.1186/s12934-016-0442-6 Bacterial strains and plasmids used in this study. [file 12934_2016_442_MOESM2_ESM.pdf]

Table S2. Bacterial strains and plasmids used in this study

Strain/Plasmids	Genotype or phenotype	Source
**Strain**		
Escherichia coli		
JM109	recA, endA1, gyrA96, hsdR17, supE44, relA1, Δ(lac-proAB)/ F′ [traD36, proAB+, lacIq, lacZΔM15]	Sangon Biotech Co.
K. pneumonia		
M5a1	wild type	This lab
Paenibacillus sp. WLY78	Wild-type nitrogen fixer	This lab.
**Plasmids**		
pHY300plk-78	pHY300plk derivative, carrying *nif* cluster driven by its original promoter, Amp^R, Tet^R	This lab
pBluescript II SK (+)	ColE1, lacZ', Amp^R	Sangon Biotech Co
pCoU	ColE1,Kan^R	Sangon Biotech Co
pBluescript II SK (+)-PnifCat	pBluescript II SK (+) derivative, carrying *nif* cluster promoter and chloramphenicol resistance cassette, Amp^R, Cat^R	This study
pBC-J	pBluescript II SK (+) derivative, carrying *nifJ* gene driven by *Paenibacillus nif* cluster promoter and chloramphenicol resistance cassette, Amp^R, Cat^R	This study
pBC-F	pBluescript II SK (+) derivative, carrying *nifF* gene driven by *Paenibacillus nif* cluster promoter and chloramphenicol resistance cassette, Amp^R, Cat^R	This study
pBC-US	pBluescript II SK (+) derivative, carrying *nifUS* genes driven by *Paenibacillus nif* cluster promoter and chloramphenicol resistance cassette, Amp^R, Cat^R	This study
pBC-WZM	pBluescript II SK (+) derivative, carrying *nifWZM* genes driven by *Paenibacillus nif* cluster promoter and chloramphenicol resistance cassette, Amp^R, Cat^R	This study
pBC-Q	pBluescript II SK (+) derivative, carrying *nifQ* gene driven by *Paenibacillus nif* cluster promoter and chloramphenicol resistance cassette, Amp^R, Cat^R	This study
pBC-fldA	pBluescript II SK (+) derivative, carrying *fldA* gene driven by *Paenibacillus nif* cluster promoter and chloramphenicol resistance cassette, Amp^R, Cat^R	This study
pBC-fer	pBluescript II SK (+) derivative, carrying *fer* gene driven by *Paenibacillus nif* cluster promoter and chloramphenicol resistance cassette, Amp^R, Cat^R	This study
pBC-fldB	pBluescript II SK (+) derivative, carrying *fldB* gene driven by *Paenibacillus nif* cluster promoter and	This study

	chloramphenicol resistance cassette, Amp^R, Cat^R	
pBC-COG3411	pBluescript II SK (+) derivative, carrying *COG3411* gene driven by *Paenibacillus nif* cluster promoter and chloramphenicol resistance cassette, Amp^R, Cat^R	This study
pBC-pfo	pBluescript II SK (+) derivative, carrying *pfoAB* gene driven by *Paenibacillus nif* cluster promoter and chloramphenicol resistance cassette, Amp^R, Cat^R	This study
pBC-nfrA	pBluescript II SK (+) derivative, carrying *nfrA* gene driven by *Paenibacillus nif* cluster promoter and chloramphenicol resistance cassette, Amp^R, Cat^R	This study
pBC-fpr	pBluescript II SK (+) derivative, carrying *fpr* gene driven by *Paenibacillus nif* cluster promoter and chloramphenicol resistance cassette, Amp^R, Cat^R	This study
pBC-suf	pBluescript II SK (+) derivative, carrying *suf* cluster genes driven by *Paenibacillus nif* cluster promoter and chloramphenicol resistance cassette, Amp^R, Cat^R	This study
pBC-isc	pBluescript II SK (+) derivative, carrying *isc* cluster genes driven *Paenibacillus nif* cluster promoter and chloramphenicol resistance cassette, Amp^R, Cat^R	This study
pCK-F	pCoU derivative， carrying *nifF* gene driven by *Paenibacillus nif* cluster promoter, Kan^R	This study
pCK-FUS	pCoU derivative， carrying *nifF* and *nifUS* genes driven by *Paenibacillus nif* cluster promoter, Kan^R	This study
pCK-fldA	pCoU derivative， carrying *fldA* gene driven by *Paenibacillus nif* cluster promoter, Kan^R	This study
pCK-fer	pCoU derivative， carrying *fer* gene driven by *Paenibacillus nif* cluster promoter, Kan^R	This study
pCK-fldAUS	pCoU derivative， carrying *fldA* and *nifUS* genes driven by *Paenibacillus nif* cluster promoter, Kan^R	This study
pCK-ferUS	pCoU derivative, carrying *fer* and *nifUS* genes driven by *Paenibacillus nif* cluster promoter, Kan^R	This study
