# Supplementary material for: Using synthetic biology to increase nitrogenase activity
Source: Microb Cell Fact. 2016 Feb 20;15:43. doi: 10.1186/s12934-016-0442-6 (PMC4761190; doi:10.1186/s12934-016-0442-6)

Figure S1. Comparison of different NifH proteins showing the Proline258 is conserved in *Paenibacillus*.

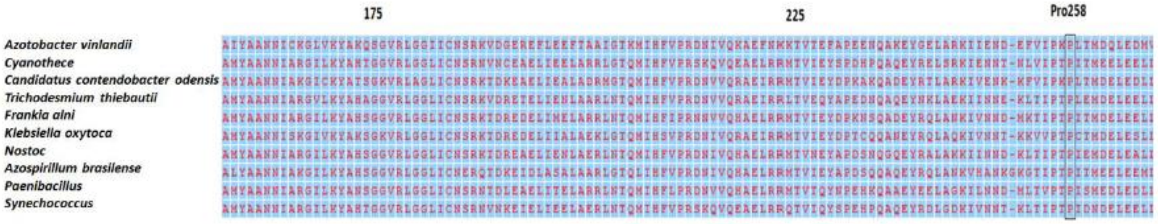

Supplement: Supplementary file 3 — 10.1186/s12934-016-0442-6 Comparison of different NifH proteins showing the Proline258 is conserved in Paenibacillus. [file 12934_2016_442_MOESM3_ESM.pdf]
