# Supplementary material for: Using synthetic biology to increase nitrogenase activity
Source: Microb Cell Fact. 2016 Feb 20;15:43. doi: 10.1186/s12934-016-0442-6 (PMC4761190; doi:10.1186/s12934-016-0442-6)
Supplement: Supplementary file 4 — 10.1186/s12934-016-0442-6 The recombinant strains constructed in this study. [file 12934_2016_442_MOESM4_ESM.pdf]

Table S3. The recombinant strains constructed in this study.

strain	plasmid	Source
E.coli 78-7(US)	pHY300plk-78 and pBC-US	This study
E.coli 78-7(suf cluster)	pHY300plk-78 and pBC-suf cluster	This study
E.coli 78-7(isc cluster)	pHY300plk-78 and pBC-isc cluster	This study
E.coli 78-7(fldA)	pHY300plk-78 and pBC-fldA	This study
E.coli 78-7(fer)	pHY300plk-78 and pBC-fer	This study
E.coli 78-7(pfoAB)	pHY300plk-78 and pBC-pfoAB	This study
E.coli 78-7(nifF)	pHY300plk-78 and pBC-nifF	This study
E.coli 78-7(nifJ)	pHY300plk-78 and pBC-nifJ	This study
E.coli 78-7(fldB)	pHY300plk-78 and pBC-fldB	This study
E.coli 78-7(COG3411)	pHY300plk-78 and pBC-COG3411	This study
E.coli 78-7(nfrA)	pHY300plk-78 and pBC-nfrA	This study
E.coli 78-7(fpr)	pHY300plk-78 and pBC-fpr	This study
E.coli 78-7(nifQ)	pHY300plk-78 and pBC-nifQ	This study
E.coli 78-7(WZM)	pHY300plk-78 and pBC-WZM	This study
E.coli 78-7(nifF)	pHY300plk-78 and pBC-US	This study
E.coli 78-7(nifJ)	pHY300plk-78 and pBC-US	This study
E.coli 78-7(FJ)	pHY300plk-78, pBC-J and pCK-F	This study
E.coli 78-7(FJUS)	pHY300plk-78, pBC-J and pCK-FUS	This study
E.coli 78-7(fldApfoAB)	pHY300plk-78, pBC-pfoAB and pCK-fldA	This study
E.coli 78-7(ferpfoAB)	pHY300plk-78, pBC-pfoAB and pCK-fer	This study
E.coli 78-7(fldApfoABUS)	pHY300plk-78, pBC-pfoAB and pCK-fldAUS	This study
E.coli 78-7(ferpfoABUS)	pHY300plk-78, pBC-pfoAB and pCK-ferUS	This study
