# Supplementary material for: Using synthetic biology to increase nitrogenase activity
Source: Microb Cell Fact. 2016 Feb 20;15:43. doi: 10.1186/s12934-016-0442-6 (PMC4761190; doi:10.1186/s12934-016-0442-6)
Supplement: Supplementary file 5 — 10.1186/s12934-016-0442-6 Primers used in this study. [file 12934_2016_442_MOESM5_ESM.pdf]

Table S4. Primers used in this study

Gene name	Forward primer (5' - 3')	Reverse primer (5' - 3')	Location /Target
nifF	KPnifF up (AAA GGATCC GACAGGAGC GCAACATGGC)	KPnifF down (AAATCTAGAGGCTACCCG TTAACGCCTACAG)	For cloning the *K. oxytoca nifF* gene to pBluescript II SK (+)
nifJ	KPnifJ-F (GCGAAGCTTATGTCCGG AAAAATGAAA ACAATGG)	KPnifJ-R (AAAGGATCCTCATGCAGC AGGAGAACTAAAG)	For cloning the *K. oxytoca nifJ* gene to pBluescript II SK (+)
nifUS	KPnifUS-F (AAATCTAGATCCCTGGAG CTGACAGCATG TGG)	KPnifUS-R (AAAGAGCTCCAGCCACCT CCGCCTTAGCCGTAAACC G)	For cloning the *K. oxytoca nifUS* gene to pBluescript II SK (+)
fldA	S6fldA-F (GCGAAGCTTATGAACTTG GGTAAAATCATG)	S6fldA-R (AAAGAGCTCATTGCGCAT ACCTATCCCTCAC)	For cloning the *Paenibacillus fldA* gene to pBluescript II SK (+)
fer	S6fer-F (GCGAAGCTTATGGCTAAA TACACTTGGGTAGA)	S6fer-R (GTTGGATCCCTTAACCTT CTTTATTGAACGGC)	For cloning the *Paenibacillus fer* gene to pBluescript II SK (+)
nfrA	S6nfrA-F (CCCAAGCTTTAATATTTGT AGGAGTGCCATG)	S6nfrA-R (CCAGGATCCCGTATATAG CTCAAATACTAC)	For cloning the *Paenibacillus nfrA* gene to pBluescript II SK (+)
pfoAB	S6pfoAB-F (AAAGGATCC GAAAAGAAGGGGGCTTAT CAC)	S6pfoAB-R (AAAGAGCTC TGATCGAGCCACTACAGTA AC)	For cloning the *Paenibacillus pfoAB* gene to pBluescript II SK (+)
suf cluster	S6SufCluster-F (AAAGGATCCCTCCTTATG TTTATGTCACTCA)	S6SufCluster-R (CCGCTCGAGCTTGGCAAT GATACTCTTGTTA)	For cloning the *Paenibacillus suf* cluster to pBluescript II SK (+)
nifQ	KPnifQ-F (AAAAAGCTTATGCCGCCG CTCGACTGGTTG)	KPnifQ-R (TTTGGATCCTTGTTTGCA GCGCCCCCTTC)	For cloning the *K. oxytoca nifQ* gene to pBluescript II SK (+)
nifWZM	KPnifWZM-F (CCCAAGCTTGAGTATGGA GTGGTTTTATC)	KPnifWZM-R (AAAGGATCCGTTTAATCT CCTCAAGCCAG)	For cloning the *K. oxytoca nifWZM* gene to pBluescript II SK (+)
Cat	Cm(pPR9TT)-F(KpnI) (ctc ggtacc ATTAGCAGAGCGAGGTAT	Cm(pPR9TT)-R(KpnI) (ctcggtacc GCGGAACCCCTATTTGTTT)	For cloning the kanamycin resistance gene

	G)		from plasmid pPR9TT to pBluescript II SK (+)
nif promoter	Pnif-F (TCGCTCGAGGCGGAGACTATTTCCCAAAAT)	Pnif-R (GTAAGCTTCTCCTCTCTACGTTATAT)	For cloning the *nif* cluster promoter from *Paenibacillus* sp. WLY78 to pBluescript II SK (+)
fpr	S6fpr-F (ATCCTCGAGTCCTTGAATGCTCAATCGTATC)	S6fpr-R (AAAGAGCTCATAGGCACACCGTAAAAAAGG)	For cloning the *Paenibacillus fpr* gene to pBluescript II SK (+)
fldB	S6fldB-F (CGCAAGCTTGATGAAAAATATACTAATCATTAACGG)	S6fldB-R (AAAGGATCCTTAATCATGTAATACGGCTTG)	For cloning the *Paenibacillus fldB* gene to pBluescript II SK (+)
COG3411	S6COG3411-F (GCGAAGCTTTTGATTAAAGGGGAAGCTATGG)	S6COG3411-R (AAAGGATCCTTATTCCGAGCTGGATTGAC)	For cloning the *COG3411*gene from *Paenibacillus* sp. WLY78 to pBluescript II SK (+)
